# Evaluating the Effectiveness of Public Health Measures During Infectious Disease Outbreaks: A Systematic Review

**DOI:** 10.7759/cureus.55893

**Published:** 2024-03-10

**Authors:** Adewale Lawrence

**Affiliations:** 1 Pharmaceutical Medicine, Bioluminux Clinical Research, Naperville, USA

**Keywords:** emergency health intervention, epidemiological assessment, intervention effectiveness, disease control strategies, infectious disease outbreaks, public health measures

## Abstract

Over the previous three decades, the incidence of infectious disease outbreaks has considerably increased and the trend is expected to increase further. Public health measures are essential for controlling and preventing emerging outbreaks of infectious illnesses. This study is aimed at evaluating the effectiveness of public health measures during infectious disease outbreaks by summarizing the outcomes from the available evidence in the literature. A systematic review was carried out through a detailed search strategy using specific keywords applied across different electronic databases, including the Science Direct, PubMed, and EMBASE databases. Studies published between 2015 and 2024 were included with a focus on cohorts, clinical trials, longitudinal studies, case-control, and quasi-experimental studies. Low-quality studies and those published before 2015 along with incorrect findings or measures were excluded. A standardized form was used for data extraction. The quality of included studies and the risk of bias were assessed through relevant techniques. The obtained data was narrative synthesized and findings were organized systematically. The reviewed studies revealed that public health measures are considerably effective against infectious disease outbreaks. The success of various measures such as social isolation, confinement measures, and public education on hygiene against different outbreaks of respiratory infectious diseases has been well-established in the literature. Moreover, the timing of intervention application plays a vital role in their success. The implementation in the early phase of an outbreak is highly effective, as it protects more people from infection and controls the overall burden of the disease. The systematic review provided valuable insights into the efficiency of public health measures in monitoring outbreaks of infectious illnesses. The main findings suggest that appropriate public health interventions are effective in controlling the incidence of contagious disease outbreaks. Ongoing research strives to investigate measures that are most effective from the perspective of public health against various transmittable diseases to prevent future outbreaks.

## Introduction and background

An outbreak of infectious disease is described as two or more related cases of the same situation or disease in which the detected number of cases goes beyond the anticipated number in a particular location over a particular period or it may involve a single case of illness caused by a significant pathogen such as viral hemorrhagic fever or diphtheria. Infectious disease outbreaks may be widespread involving cases either regionally, nationally, or universally, or may be confined to the members of a single family [1,2]. In the previous three decades, the incidence of reported infectious disease outbreaks has been considerably increased. This trend is anticipated to continuously rise with more zoonotic spillover events (pathogens transmission from animals to humans) due to rapid population expansion and the adverse impacts of climate change [2]. The recent large outbreaks of highly transmissible or highly pathogenic infectious illnesses include diphtheria (Bangladesh), plague (Madagascar) [2], Ebola (West Africa and the Democratic Republic of Congo), SARS-CoV (severe acute respiratory syndrome coronavirus) [3], MERS-CoV (Middle East respiratory syndrome coronavirus, Korea and Saudi Arabia) [4], and Zika (Central and South America), monkeypox (Nigeria) [2]. SARS-CoV and MERS-CoV are the two extremely pathogenic HCoVs (human coronaviruses) of the twenty-first century causing global epidemics. Yet another highly infectious HCoV, a novel coronavirus, emerged recently in 2019 in Wuhan, China, which caused serious illness and mortality worldwide [5].

From the public health perspective, the primary tools available for controlling the transmission of contagious illnesses are the public health measures or interventions that have been employed for several years [6]. These measures are called public health and social measures (PHSM) and are denoted as the non-pharmaceutical measures carried out by governments, communities, and individuals to protect the well-being and health of communities impacted by health emergencies. Public health measures lower the scale and risk of the spread of infectious diseases by lowering transmission-related exposures or making them safer [7]. To prevent the transmission of infectious diseases, public health principally focuses on early detection through contact tracing and testing, along with the prevention of further spread by containment measures, for example, lockdowns, curfews, stay-at-home orders, quarantine of exposed cases [8,9], trade or travel constraints [9]. Moreover, behavioral measures including personal protective interventions (e.g. respiratory etiquettes and hand hygiene), as well as physical or social distancing measures (like workplace measures, school measures, isolation of sick persons, avoiding crowds, etc.), may also be implemented at the community level as a part of public health measures to flatten the outbreak curve [10].

The usefulness of public health measures has been indicated by the fact that contagious diseases, by the mid-twentieth century, had been considerably controlled before the use of vaccines and antibiotics became widespread [6]. The main impact of such measures in controlling the outbreaks of transferrable diseases is through the reduction of human-to-human transmission of pathogens [11]. There is enough evidence regarding the efficiency of public health strategies against different outbreaks of infectious illnesses including influenza [12], SARS [13], and COVID-19 [14-17]. Particularly, regarding the recent pandemic COVID-19, public health or non-pharmaceutical measures proved to be the most effective available measures for the regional and international control and extenuation of the infection given the lack of effective antiviral and antibiotic treatments. The measures that considerably contributed to lowering the emergence of new cases as well as the spread of the virus included isolation of existing/confirmed cases, quarantine of exposed individuals, and travel restrictions [18]. In addition to these, various other public health measures showed a significant impact against the COVID-19 outbreak in different countries including Denmark [19], South Korea [20], India [21], the United States, China, Spain, Iran, and Turkey [5,22]. Despite the availability of evidence, public health measures against infectious illness outbreaks are often criticized for causing unwanted negative consequences for individuals and communities and are questioned in terms of their effectiveness in controlling the outbreaks [7]. Given this, the current study is conducted to summarize the available evidence in support of the effectiveness of public health intervention against the outbreaks caused by infectious diseases to provide insights into the usefulness of these measures in controlling and mitigating outbreaks.

The systematic review evaluated studies published between 2015 and 2024 to provide a current assessment of the efficacy of public health measures during infectious disease outbreaks. This timeframe was most likely chosen to reflect the most recent advances in research, techniques, and the changing landscape of infectious illnesses during the last three decades. Furthermore, studies published after 2015 may take into account the global impact of the COVID-19 pandemic, emphasizing the relevance of integrating findings from this key time period. The implementation of this time frame facilitates methodological consistency, aligning the research under assessment and allowing for a more comprehensive understanding of the efficacy of public health interventions in the face of rising infectious risks.

## Review

Methods

The research question to be addressed in this systematic review was: “Whether public health measures are effective against infectious disease outbreaks?” A detailed search strategy was developed involving the use of database-specific key terms and appropriate keywords related to “public health measures”, “public health interventions”, “effectiveness”, and “infectious disease outbreaks”. The mentioned keywords were combined to generate various search combinations using Boolean operations including “AND” and “OR”.

Inclusion Criteria

These included studies evaluating the impact of non-pharmaceutical or public health measures such as contact tracing, quarantine, wearing masks, lockdown, social distancing, travel or mobility restrictions, or isolation of cases. Cohort studies, quasi-experimental studies, clinical trials, case-control studies, and longitudinal studies were also included. Only those articles that were published in the English language and were published between 2015 and 2024, addressing the recent trends, were included in this study.

Exclusion Criteria

These included studies not investigating public health interventions and reporting irrelevant outcomes, i.e. not related to infectious diseases, and studies like narrative reviews, meta-analyses, systematic reviews, case reports, cross-sectional studies, case studies, editorials or letters, and conference reports. Articles published before 2015 were not included. All articles available in languages other than English were excluded.

Search and Selection of Databases

The search strategy was implemented across different electronic databases, including Science Direct, PubMed, and EMBASE. Titles and abstracts were screened to identify the eligibility of the studies in relevance to the review question and the inclusion criteria. Highly relevant studies were selected through full-text screening followed by the evaluation of suitability based on the eligibility criteria.

Extraction of Data

A standardized form was used for data extraction to collect relevant information from each of the selected studies. Extracted data focused on research characteristics such as study design, year, types of public health measures, type of contagious diseases, and effectiveness of implementing public health measures in controlling the outbreaks.

Quality Assessment

Relevant tools were used for assessing the quality of studies along with the risk of bias. The methodological quality and potential sources of bias in each study were evaluated.

Data Synthesis and Analysis

The extracted data were synthesized and analyzed narratively, and the outcomes related to the effectiveness of public health measures against infectious disease outbreaks were organized systematically. Sensitivity and subgroup analyses were performed where applicable.

Interpretation of Outcomes

The findings were interpreted according to the limitations and strengths of the selected studies. Practical implications of findings, possible gaps in knowledge, and suggestions for future work were discussed.

Report Write-Up

**Figure 1:**
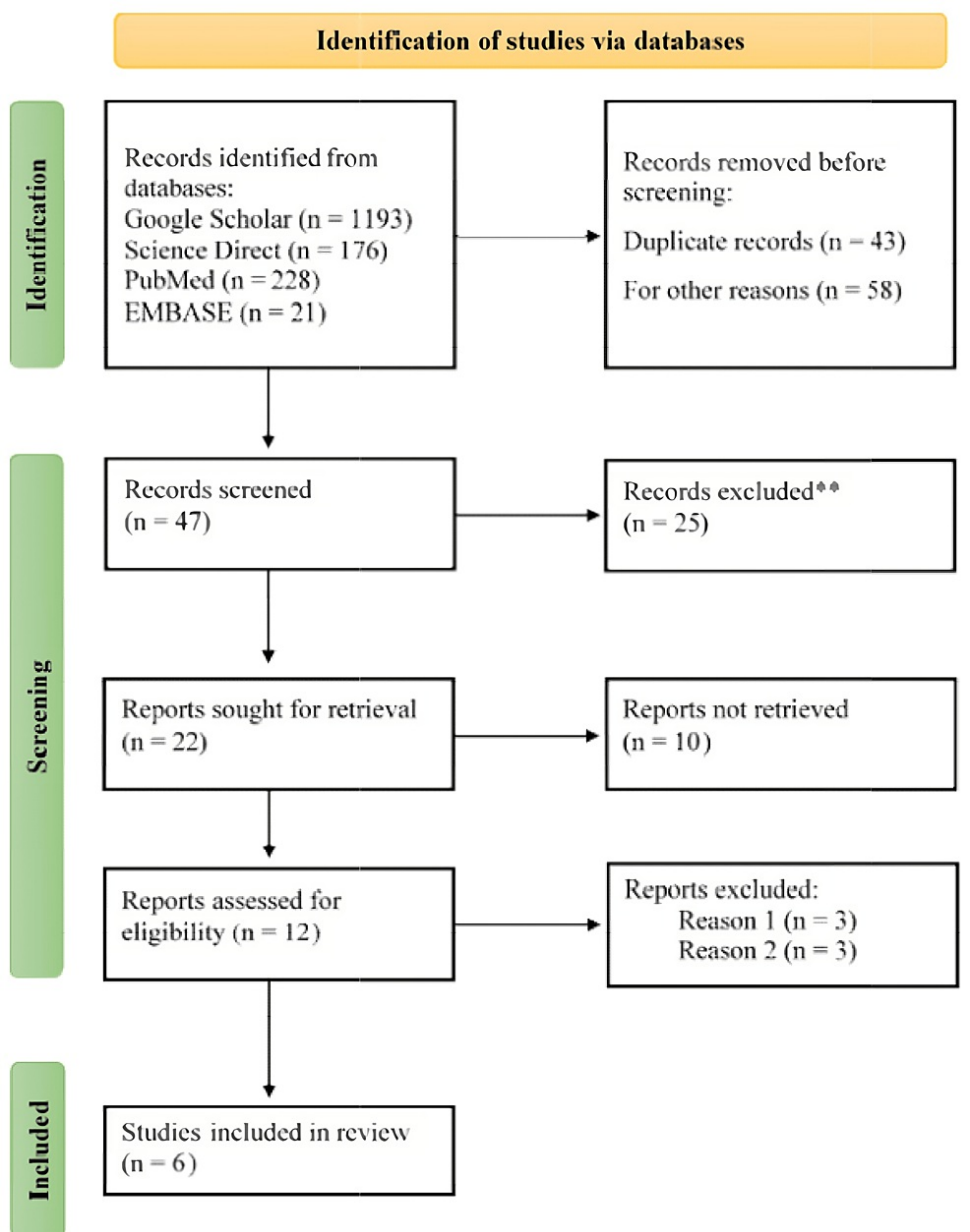
PRISMA flow chart for systematic review PRISMA: Preferred Items for Systematic Review and Meta-Analysis

The manuscript was developed following the guidelines of the PRISMA (Preferred Items for Systematic Review and Meta-Analysis) model (Figure 1). The paper was organized using distinct headings including Introduction, Methodology, Results, Discussion, and Conclusion.

Results

Table 1 explains the details of the excluded studies from the review on the effectiveness of public health measures during the COVID-19 pandemic, along with the reasons for exclusion. These exclusions range from empirical studies based on narrative reviews, review-based studies, and systematic review-based studies to those that are outdated, irrelevant to the study's outcomes, or focused on unrelated topics such as economic burden or digital tools. The criteria for exclusion were applied to ensure the relevance and quality of studies included in the review, focusing on recent and pertinent research directly related to the impact of public health interventions on controlling the spread of COVID-19.

**Table 1:** Excluded studies (author name, title, and reason for exclusion)

Sr. No.	Author(s) name and year	Title	Reasons for exclusion
1	Ge et al. 2023 [23]	Effects of public health measures for zeroing out different SARS-CoV-2 variants	An empirical study based on a narrative review of the literature
2	Shen et al. 2021 [24]	Monitoring non-pharmaceutical public health interventions during the COVID-19 pandemic	Review-based study
3	Wang et al. 2021 [25]	Evaluating the effectiveness of control measures in multiple regions during the early phase of the COVID-19 pandemic in 2020	Systematic review-based study
4	Vardavas et al. 2021 [26]	Cost-effectiveness of emergency preparedness measures in response to infectious respiratory disease outbreaks: a systematic review and econometric analysis	Systematic review-based study, irrelevant study measures and outcomes related to the economic burden of infectious diseases and public health measures
5	Talic et al. 2021 [27]	Effectiveness of public health measures in reducing the incidence of COVID-19, SARS-CoV-2 transmission, and COVID-19 mortality: systematic review and meta-analysis	Meta-analysis or systematic review-based study
6	Zeng et al. 2020 [28]	The use of digital tools to mitigate the COVID-19 pandemic: comparative retrospective study of six countries	Irrelevant outcomes report findings associated with the effectiveness of using digital tools on the application of public health measures for the control of COVID-19 spread
7	Wessel et al. 2011 [29]	Public health interventions for epidemics: implications for multiple infection waves	The old study, based on the narrative literature review
8	Lee et al. 2010 [30]	Effectiveness of public health measures in mitigating pandemic influenza spread: a prospective seroepidemiological cohort study	Old study
9	Fraser et al. 2004 [31]	Factors that make an infectious disease outbreak controllable	Too old study, irrelevant outcomes, investigates factors that affect the effectiveness of public health measures to control infectious illness outbreaks
10	Jajosky and Groseclose 2004 [32]	Evaluation of reporting timeliness of public health surveillance systems for infectious diseases	Too old study, based on a systematic review of literature, irrelevant in terms of evaluation of the performance of surveillance systems related to public health during outbreaks of infectious diseases

Table 2 explains that the longitudinal study by Hu et al. investigated the correlation between public health measures implemented during the pandemic COVID-19 and the incidence of various contagious diseases of the respiratory tract in China. They reported that while public health measures of social distancing, isolation, and wearing masks reduced the spread of COVID-19, these interventions resulted in significant reductions in the incidences of other six infectious respiratory illnesses including influenza, tuberculosis (TB), scarlet fever, measles, pertussis, and mumps. These measures particularly led to the disappearance of the seasonal elevations in the occurrence of these six infections in the years 2020 and 2021. This implies that public health measures were found to be more effective against these illnesses during the months in which seasonal occurrence usually heightened. Furthermore, among all the six infections, TB was the only one, which was least influenced by the measures. Overall, public health strategic measures effectively lowered the frequency of transmittable illnesses of the respiratory tract during the period when they would normally have peaked [33]. The investigation done by Lee et al. evaluated public health strategic measures carried out during the period of the COVID-19 outbreak to control the outbreak as well as lower the incidence of the influenza epidemic in South Korea. A variety of public and social health measures were implemented, including compulsory public education on cough etiquette, hand hygiene, universal mask-wearing, staying at home, avoiding unnecessary crowding in social activities, school closures, etc. These measures not only led to a reduction in the spread of COVID-19 but also considerably lowered the seasonal influenza epidemic from 2019 to 2020 by 6 to 12 weeks. In addition, the influenza peak reduced to 49.8 ILIs (influenza-like illness) per 1000 visits as compared to 71.9-86 ILIs per 1000 visits in the previous season. Moreover, influenza hospitalization cases also lowered by 11.9 to 26.9 fold, and influenza B cases reduced from 26.6-54.9% in 2019 to 4% of all cases in 2020. These results imply that public health measures used during COVID-19 may assist as effective strategies for controlling and preventing influenza epidemics in the coming seasons [34]. Matrajt and Leung examined the value of social distancing public health measures in controlling the COVID-19 outbreak in a mid-sized city in the United States in 2020. As per the reporting outcomes, the implementation of interventions resulted in decreased contacts for six weeks among different age groups including children below or equal to the age of 19, adults between the ages of 20 and 59 years, and adults more than or equal to 60 years of age. In addition, the results also revealed that the timing of intervention implementation also played a considerable role in their effectiveness against the outbreak. Earlier implementation of interventions in the preliminary phase of the epidemic delayed the epidemic curve, whereas, later implementation flattened the curve. Moreover, these measures also averted new cases, hospitalizations, as well as deaths reported among the adult population. The study concluded that social distancing measures can enhance healthcare capacity against infectious disease transmission [35]. The longitudinal study of Rocklöv et al. evaluated the usefulness of public health measures against the COVID-19 outbreak that occurred on the Diamond Princess cruise ship in February 2020. The measures included isolation and removal of sick passengers and quarantine of non-sick passengers. Findings revealed that the basic reproduction rate on board was four times higher initially than that in the outbreak epicenter in Wuhan, China; however, the implementation of public health measures significantly lowered this rate. More specifically, quarantine and isolation prevented more than 2000 new cases by lowering the rate from 14.8 (initially without interventions) to 1.78 after countermeasures. The study suggested that early removal of passengers from the ship would have prevented many more cases from emerging as the conditions of the cruise ship amplified the cases in the absence of interventions [36]. Wong et al. conducted an evaluation of the effect of national containment public health measures, including lockdowns, curfews, and stay-at-home orders, implemented across 54 countries and four epicenters (Wuhan, Madrid, New York State, and Lombardy) of the COVID-19 outbreak in 2020. The containment measures were implemented in countries that reported a 10%, 20%, and 30% increase in new cases daily. The findings of the study revealed that a lowering trend in the percent increase in new cases/day was observed within seven days of the commencement of all three interventions. In addition, the percent increase in daily new cases reduced to less than five within one month. These findings imply that confinement measures proved to be effective public health strategic measures against outbreaks of the COVID-19 infectious disease [9]. The investigation by Salvatore et al. assessed the efficiency of lockdown as a public health measure against the spread of COVID-19 in India in the year 2020. Results reported a reduction in the basic reproduction rate from 3.36 (CI 95% 3.03 - 3.71) to 1.27 (CI 95% 1.26 - 1.28) before and after the implementation of the lockdown measure, respectively. In addition, delays in the doubling time from 3.56 days to 14.37 days were also reported. However, the average number of tests daily increased (from 1717 to 113,372) along with an increase in test positivity rate (from 2.1 to 4.2%). These patterns of changes over the lockdown commencement period imply that public health-related measures were partially useful in lowering the transmission of corona in India [37].

**Table 2:** Studies included in the review

Sr. No.	Author(s) name and year of publication	Study design	Infectious disease(s) outbreaks	Public health measures	Findings
1	Hu et al. 2021 [33]	Longitudinal study	COVID-19	Physical distancing, mask-wearing, isolation	Substantial impact in controlling the COVID-19 outbreak as well as the frequency of other contagious illnesses of the respiratory tract including scarlet fever, measles, mumps, influenza, tuberculosis, and pertussis in 2020 with respective P values of 0.002, 0.002, 0.002, 0.034, 0.002, and0.004.
2	Lee et al. 2021 [34]	Cohort study	COVID-19, seasonal influenza epidemic	Public education on cough etiquette, hand hygiene, mask usage, stay-at-home orders, school closures, avoidance from unnecessary social activities	Significant decrease in COVID-19 as well as the activity of seasonal influenza. Significant reduction in influenza B cases in 2019/2020 accounting for 4% of all cases in 2020 as compared to 26.6 to 54.9% in 2019.
3	Matrajt and Leung 2020 [35]	Cohort study	COVID-19	Social distancing measures	Earlier implementation of interventions resulted in delaying the epidemic curve, while later implementation flattened the curve indicating considerable effectiveness in controlling the COVID-19 outbreak.
4	Rocklöv et al. 2020 [36]	Longitudinal study	COVID-19	Quarantine, isolation, early evacuation	Remarkable reduction in the basic reproduction rate after the implementation of countermeasures. The initial reproduction rate of 14.8 was reduced to 1.78 which prevented more than two thousand new cases in comparison to no measures.
5	Wong et al. 2020 [37]	Longitudinal study	COVID-19	National containment measures including lockdowns, stay-at-home orders, curfews	Measures significantly decreased new cases of confirmed infection lowering the % rise in daily new cases to less than five in a single month.
6	Salvatore et al. 2020 [38)	Retrospective cohort study	COVID-19	Four-phase national lockdown	Partial effectiveness of measures in controlling the transmission of COVID-19 infection. Reduction in reproduction rate from 3.36 (CI 95% 3.03 – 3.71) to 1.27 (CI 95% 1.26 – 1.28). Delay in doubling time from 3.56 days to 14.37 days.

Risk of Bias (ROB) of Studies Included in the Review

Different ROBs may arise in the included articles for a variety of reasons. The study by Hu et al. included patients suffering from only six respiratory transferrable illnesses, including influenza, tuberculosis, mumps, pertussis, scarlet fever, and measles. Therefore, selection bias may occur in this study, and the findings may not be generalized to patients suffering from other respiratory infections in China. Lee et al.’s study included data on laboratory-confirmed cases of COVID-19 from the official websites of South Korea. Thus, the non-reported cases were not included in the study, which may have resulted in selection bias. Matrajt and Leung’s investigation considered mild and severe cases of COVID-19 to be equally infectious, which may have resulted in outcome reporting bias. Rocklöv et al. investigated the confirmed cases of COVID-19 outbreak from a single cruise ship in Japan, so the results may not be used to represent the entire population in Japan. The study of Wong et al. included a limited sample of 122,366 COVID-19 patients from 54 countries, therefore, their findings may also not be representative of the entire population of people in these countries. Salvatore et al.’s study investigated cases from 20 states of India. So, the results may not be generalized to the entire population in India. It is worth noting that some possible biases may influence the findings of studies included in the current systematic review. The particular ROB for individual studies may differ based on the characteristics of participants, methodology, or the research design employed.

Discussion

Full-text screening of the studies included in this review was aimed at evaluating the usefulness of implementing public health measures in the control and prevention of outbreaks of infectious diseases. Given this, the study gathered and summarized evidence from the published literature supporting the usefulness of public health interventions against the epidemics/ pandemics of contagious illness. Researchers in the reviewed studies sought to provide valuable data about the relationship of public health measures with a reduction in the frequency of various contagious diseases and their outbreaks by conducting cohort and longitudinal studies across different countries. The effectiveness of public health strategic measures in controlling infectious disease outbreaks is demonstrated by their impact on lowering the transmission rates of infectious agents by minimizing the contact between healthy and ill persons. The main outcomes suggested that the execution of national and universal public health measures is an effective strategy to reduce the spread of infectious illness outbreaks locally, nationally as well as globally. The majority of reviewed studies investigated the efficiency of different public health interventions in controlling and mitigating the recent outbreak of COVID-19. Outcomes highlighted that enforcing social distancing and public education measures at national and global levels effectively lowered the spread of the disease by reducing the occurrence of new cases, rate of hospitalization, and mortality from the infection across various countries. Moreover, these measures also proved to be useful against other infectious diseases, including influenza, measles, tuberculosis, mumps, scarlet fever, etc. In addition, it is found that developing appropriate measures and timing is important for their successful implementation where early implementation is highly effective in controlling the outbreak from getting out of hand. Consequently, this systematic review put forward that the effective and timely application of appropriate public health measures is substantially associated with the successful control and prevention of infectious disease outbreaks.

Similar results have been reported by other studies. Zhao et al. reported the usefulness of social distancing and other public health interventions in lowering the incidence of reported cases of COVID-19 by 99.99% after the comprehensive implementation in Jilin province of China [14]. Aledort et al. demonstrated that enforcing measures, such as respiratory/cough etiquette, hand hygiene, self-isolation, and the proper use of personal protective equipment, including masks, is effective in controlling the influenza pandemic [12]. Bell also reported the effectiveness of increased social distancing, isolation of case patients, and quarantine measures in controlling the SARS pandemic [13].

Overall, the reviewed studies emphasize the significance of public health measures during infectious disease outbreaks. The evidence obtained from these studies is of high importance for notifying healthcare policymakers, clinical practitioners, and global and local healthcare authorities regarding the timely implementation of appropriate public health measures for controlling the occurrence of various infectious disease outbreaks. This data can help in tailoring and improving the existing public health measures and control strategies to prevent outbreaks of infectious illnesses in the future.

Although the studies provided valuable insights into the usefulness of public health interventions during infectious illness outbreaks, certain limitations must be considered: The majority of studies focused on public health measures against the COVID-19 pandemic, so their findings are limited regarding other infectious diseases [35-37]. In addition, none of the studies, except one, explored the age or gender-based subgroups, therefore, the findings cannot be generalized to the entire population or those at risk [35]. Moreover, these studies did not provide evidence about the measures that could be most effective in improving public health response to control infectious disease outbreaks. It is essential to consider these limitations in future research to provide more reliable and comprehensive results about the effectiveness of public health interventions against the successful control of infectious disease outbreaks.

Interpretation of Main Results as Physiological Plausibility

The main results of this systematic review regarding the effectiveness of public health strategic measures against outbreaks of communicable diseases are physiologically plausible. Rocklöv et al. and Salvatore et al. have reported a reduction in the reproduction rate of the SARS-CoV-2 virus after the implementation of countermeasures [36,37]. This is explained by Wong et al. who have mentioned that one of the main modes of transmission for the COVID-19 virus is respiratory droplets [9]. Thus, decreasing the population movement and social contact through social distancing measures helps avert the spread of the outbreak. While Hu et al. and Lee et al. reported a reduction in the prevalence of various other infectious diseases along with COVID-19 [33,34]. Ayouni et al. have also reported those non-pharmaceutical interventions, such as social distancing, mask-wearing, public education on hygiene, etc., lower the chances of contact with the infectious agent and thus, play an effective role in controlling outbreaks and maintaining suppression [5].

Clinical Plausibility of Results

The findings support the clinical plausibility of the efficiency of public health interventions in preventing infectious disease outbreaks. The timing of implementing public health countermeasures is of critical importance. Starting interventions in the early stage of an outbreak can be highly operational in controlling the spread of the infectious agent by lowering the incidence of new cases, rate of hospitalizations, and mortality [35]. This will eventually lower the burden on clinical and healthcare facilities, enabling them to focus their attention on the confirmed cases. Furthermore, clinical and health care practitioners have a crucial role in this regard by informing and encouraging the public about non-pharmaceutical measures through proper guidance and counseling to successfully implement these measures and prevent the further transmission of outbreaks.

Implication of Main Outcomes

The implications of the main findings indicate that public health measures are useful in stopping the spread of outbreaks of infectious diseases. In particular, the timely execution of social and public health measures (in the initial stage of the outbreak) considerably contributes to controlling the disease spread by delaying or flattening the epidemic curve [34]. In addition, devising appropriate public health measures keeping in view the characteristics of the infectious agent is of high importance to effectively control a particular outbreak [33]. Moreover, tailoring public health interventions while considering the needs of the general public along with the overall social and economic costs can make such measures more effective preventive strategies against infectious diseases [26,37].

Future Perspectives

Future research needs to focus on conducting comprehensive studies on different outbreaks or epidemics of infectious diseases in different countries. This is because the population structures and the health care infrastructures vary from country to country, and thus the effects of public health measures could also differ widely in different regions. Therefore, it is essential to identify which public health measures would be most effective in controlling specific infectious disease outbreaks globally as well as in particular regions. Furthermore, exploring more subgroups, such as age, gender, education level, income distribution, etc., would also provide useful information about the factors that may hinder or promote the effectiveness of public health measures against infectious illness outbreaks.

Limitations

The limitations of the study were that it only focussed on the COVID-19 outbreak and some respiratory disease epidemics. It does not provide data on subgroups (age, gender, etc.). It does not consider the long-term economic and social costs of public health measures. It also lacks long-term follow-up assessment plans.

Recommendations

Recommendations for future studies are that the researcher should conduct comprehensive studies evaluating the effectiveness of public health measures against various outbreaks of infectious diseases in different geographical areas to provide reliable conclusions and focus on the identification of public health measures that are most effective against a particular infectious illness outbreak. Moreover, they should comprehensively explore particular subgroups, including long-term assessment plans or duration, and assess the cost-effectiveness as well.

## Conclusions

To conclude, the current systematic review provided valuable insights into the effectiveness of public health measures in controlling outbreaks of infectious illnesses. The main findings from the reviewed studies suggest that appropriate public health interventions are effective in controlling the incidence of infectious disease outbreaks. Various public health interventions, such as social distancing, confinement measures, and public education on hygiene, are considerably effective against different outbreaks of respiratory infectious diseases. Moreover, the timing of intervention implementation plays a vital role in their success. The implementation in the early stage of the outbreak is highly effective, as it protects more people from infection and controls the overall burden of the disease. The ongoing research from the perspective of public health strives to investigate measures that could be highly effective against various contagious diseases to prevent future outbreaks.
